# HybridNER: A Multi-Model Ensemble Framework for Robust Named Entity Recognition—From General Domains to Adversarial GNSS Scenarios

**DOI:** 10.3390/s26051553

**Published:** 2026-03-02

**Authors:** Yixuan Liu, Jing Zhang, Ruipeng Luan, Xuewen Yu

**Affiliations:** Academy of Military Science, People’s Liberation Army, Beijing 100850, China; liu3712025201@163.com (Y.L.);

**Keywords:** named entity recognition, ensemble learning, satellite navigation countermeasures, large language models

## Abstract

Named entity recognition (NER), a core task in natural language processing (NLP), remains constrained by heavy reliance on annotated data, limited cross domain generalization, and difficulty in recognizing name entities out of vocabulary entities. In specialized domains such as analysis of Global Navigation Satellite System (GNSS) countermeasures, including anti-jamming and anti-spoofing, where datasets are small and domain knowledge is scarce, existing models exhibit marked performance degradation. To address these challenges, we propose HybridNER, a framework that integrates locally trained span-based models with large language models (LLMs). The approach employs a span prediction metasystem that first fuses outputs from multiple base learners by computing span to label compatibility scores and assigns an uncertainty estimate to each candidate entity. Entities with uncertainty above a preset threshold are then routed to an LLM for a second stage classification, and the final decision integrates both sources to realize complementary strengths. Experiments on multiple general purpose and domain specific datasets show that HybridNER achieves higher precision, recall, and F1 than traditional ensemble methods such as majority voting and weighted voting, with especially pronounced gains in specialized domains, thereby improving the robustness and generalization of NER.

## 1. Introduction

Named Entity Recognition (NER) [1] is a foundational task in Natural Language Processing (NLP) that involves identifying named entities in text corpora and assigning them to predefined categories. These entities are typically words or phrases that denote real-world objects such as persons, times, and locations. For example, in the sentence “In 1973, the United States began developing the Global Positioning System,” “1973” is annotated as a Time entity and “the United States” as a Location entity. Accordingly, NER performs both detection and classification of entities in text, with coverage ranging from proper nouns to complex concepts, and it constitutes a core component of applications including machine translation, question answering, and information retrieval [2]. The methodology of NER has evolved from rule-based approaches to pre-trained language models. Early methods relied on handcrafted features; although they offered high interpretability, they exhibited weak generalization. The rise of machine learning enabled data-driven modeling, while neural networks with hierarchical architectures automatically capture deep semantic representations. More recently, large language models (LLMs), benefiting from massive parameterization and strong context modeling, have delivered significant advances in fine-grained entity recognition and cross-domain transfer learning, thereby elevating NER to a new level of performance [3].

Recent studies show that NER models encounter substantial challenges when applied to specialized domains. These difficulties are frequently observed in social media and clinical text, and they are particularly pronounced in highly specialized settings such as satellite navigation countermeasures. The central cause is the typically small size of domain-specific datasets, which prevents models from adequately acquiring domain knowledge during training and leads to marked declines in both detection accuracy and classification performance for specialized entities. Consequently, even with targeted fine-tuning, compact NER models remain limited by insufficient domain knowledge, which constrains their practical effectiveness in specialized scenarios. To address these limitations, the research community has explored two main directions. The first injects external knowledge by leveraging domain-specific knowledge bases to enrich entity vocabularies and improve recognition accuracy. This approach, however, faces clear constraints: coverage is available only in a few domains with mature resources, and the construction and maintenance of such knowledge bases require substantial human and temporal costs. In the medical field, for example, the Unified Medical Language System (UMLS) [4] comprises roughly two million medical concepts and more than five million lexical entries, yet comparable high-quality resources remain scarce in most specialized areas. The second direction focuses on optimizing data-distribution mismatch by modeling and adapting to domain-specific distributions, thereby reducing overreliance on the entity distribution present in the training data and improving recognition of unseen entities [5]. Unfortunately, the real-world performance of such methods is generally modest and still falls short of the usability and reliability required in specialized applications. In sum, current NER models do not yet meet the operational demands of specialized domains, highlighting the need for further methodological advances.

The emergence of LLMs has marked an inflection point for artificial intelligence. Pretrained on massive corpora, LLMs acquire rich linguistic representations and domain knowledge, yielding stronger comprehension and generalization that have propelled advances across numerous natural language processing tasks and substantially influenced NER [6]. However, despite their strengths in contextual understanding and text generation, their performance on NER remains comparatively limited. A plausible explanation is that LLMs are not explicitly trained for NER and lack a clear inductive bias for span boundary detection and entity type assignment, reflecting insufficient task-specific specialization [7]. In addition, their enormous parameterization and, in many cases, restricted access to model weights impede task-adaptive fine-tuning and targeted improvement in domain expertise. Consequently, relying solely on LLMs to handle NER in specialized domains remains a significant challenge.

**Figure 1:**
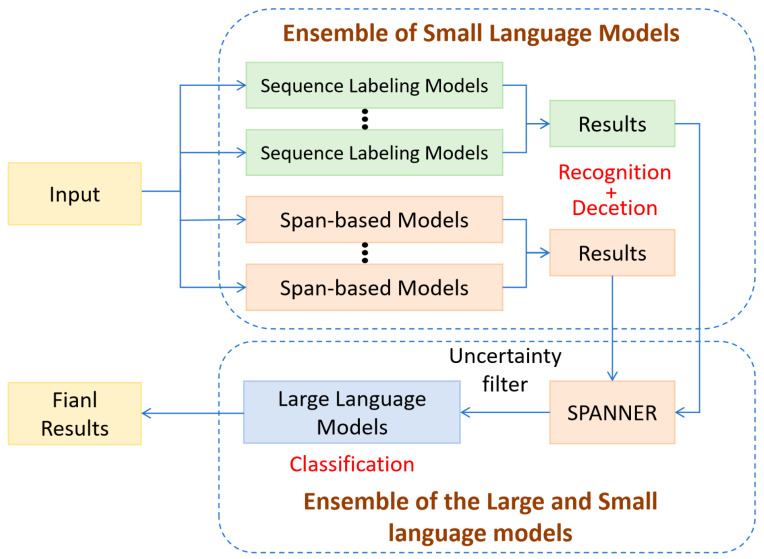
HybridNER framework.

To address the foregoing challenges, we introduce HybridNER, a collaborative framework that links domain-adapted local named entity recognition models with black box large language models in order to strengthen robustness across diverse entity types. First, the local models undergo domain adaptation through targeted fine tuning. Contemporary approaches to named entity recognition fall into two paradigms: sequence labeling and span level prediction. The former is well suited to long entities and to cases with weak tag consistency, whereas the latter tends to perform better on unseen entities and on entities of moderate length. Guided by these observations, we adopt SpanNER as a meta system that aggregates the outputs of multiple base systems and integrates the strengths of the two paradigms to achieve complementary capabilities. This design allows the local models to realize their full recognition potential while assigning calibrated uncertainty scores to candidate entities, which enables precise identification of difficult cases for subsequent verification. In the verification stage, the recognition of difficult entities is reformulated as a classification problem and handled by large language models for a second stage decision; the final prediction fuses the outputs of the local models and the large language models. In this process, the generalization capacity of large language models compensates for knowledge gaps in the local models, while the domain specific distributional patterns learned by the local models mitigate the lack of specialization in the large language models, producing a synergistic effect. We validate the framework through controlled experiments on several specialized datasets and observe consistently superior results, indicating that HybridNER is broadly applicable to named entity recognition in specialized domains. The architecture of the framework is illustrated in Figure 1.

The contributions of this paper are summarized as follows:

(1) We propose HybridNER, a hybrid named entity recognition framework that integrates sequence labeling, span-based prediction, and large language models. By fusing outputs and leveraging complementary strengths, the framework enhances both entity boundary detection and entity type classification.
(2) We conduct a systematic evaluation on Chinese general-purpose and domain-specific corpora, demonstrating effectiveness and stability across diverse genres and task settings.
(3) We construct a dataset for satellite navigation countermeasures, specifically Global Navigation Satellite System anti-jamming and anti-spoofing, and show that HybridNER delivers strong performance and robust generalization in knowledge-intensive scenarios characterized by specialized terminology, long tail distributions, niche domain coverage, and strong dependence on background knowledge.

## 2. Materials and Methods

### 2.1. Task Definition

This section formalizes the task of NER and outlines the core objectives and methodological pathway of the HybridNER framework. The objective of NER is to automatically identify named entities of predefined categories from a given text sequence and to determine both entity boundaries and entity types. HybridNER addresses this task by integrating two modeling paradigms, sequence labeling and span level prediction, within a collaborative scheme that couples locally fine-tuned domain-adapted models with LLMs. The framework is designed to tackle three challenges that are prominent in Chinese domain-specific NER: ambiguous entity boundaries, difficulty in recognizing specialized terminology, and weak generalization to out-of-vocabulary items. By combining local adaptation with selective verification using LLMs, HybridNER aims to achieve simultaneous improvements in robustness and recognition accuracy.

#### 2.1.1. Formal Description of the NER Task

Given an input text sequence $X={\{x}_{1},x_{2},...,x_{n}\}$ (where $x_{i}$ represents the i-th token in the sentence), the NER task outputs predictions that include entity boundary and type information, typically implemented via two mainstream modeling approaches [8]. In sequence labeling models [9], an output label $y_{i}$ is assigned to each token $x_{i}$ in the input sequence, yielding a label sequence $Y={\{y}_{1},y_{2},...,y_{n}\}$. The labeling scheme follows the widely used BIO tagging convention, where “B” marks the beginning of an entity, “I” marks the inside of an entity, and “O” marks a non-entity position; prefixes distinguish entity types (e.g., “B-PER” indicates the beginning of a person entity, “I-LOC” indicates the inside of a location entity). Under this paradigm, entity boundaries and types can be parsed from the label sequence, and it is suitable for scenarios in which entity boundaries are relatively aligned with token boundaries. The span-prediction model [10] first enumerates all possible contiguous text spans in the sequence to form a candidate span set $S=\{s_{1},s_{2},...,s_{m}\}$ (where m denotes the total number of candidate spans), with $s_{i}=\{x^{e},x^{e+1},...,x^{d}\}$ representing the contiguous segment from the start token $x^{e}$ to the end token $x^{d}$. It then assigns an entity-type label l to each candidate span $s_{i}$ and computes the uncertainty value u of the prediction; the final output format is $y=[(x^{e},x^{d},label),u]$. This paradigm models the entire entity span directly without relying on token-level label dependencies, and it is more advantageous for recognizing nested entities, overlapping entities, and out-of-vocabulary (OOV) entities.

#### 2.1.2. HybridNER Task Objectives and Evaluation Metrics

The core task objectives of the HybridNER framework are organized into two tiers. The first tier is local model ensemble optimization: the SpanNER meta-system integrates the outputs of sequence labeling and span-prediction models, uses a span-label compatibility score to complement the strengths of the two paradigms, assigns an uncertainty value u to each predicted entity, and flags high-uncertainty entities. The second tier is cross-model collaborative optimization: the flagged difficult entity-recognition cases are reformulated as an entity classification task; leveraging the knowledge-completion capability of LLMs, a second-stage classification is performed. Finally, the outputs of the local models and the LLMs are integrated to produce entity-recognition results that jointly exhibit “data-distribution adaptability” and “coverage of domain expertise”. To objectively evaluate model performance, this study adopts the standard metrics in information extraction, namely precision, recall, and F1 score, as the core evaluation criteria. The F1 score is the harmonic mean of precision and recall, providing a balanced assessment of the model’s ability to control false positives and false negatives.

### 2.2. Datasets

To verify the adaptability of the HybridNER framework in both general domains and specialized domains, this study selects four Chinese NER datasets for experimentation. Only MSRA [11] belongs to the general domain, whereas CLUENER [12], CMeEE [13], and a self-constructed Satellite Navigation Countermeasures (SNCM) dataset belong to specialized domains. The information for each dataset and its usage in the experiments are as detailed below.

#### 2.2.1. General Domain Dataset

The MSRA dataset was released by Microsoft Research Asia in 2006 and is a classic benchmark for Chinese NER. Its corpus is sourced from news reports and covers general topics such as politics, finance, and sports. Entity annotation follows the BIO scheme and includes three core entity types: person, location, and organization. The official standard split comprises 46,364 sentences for training, 3442 sentences for validation, and 3683 sentences for testing. In this study, we adopt the community-standard preprocessed version, preserving the original annotations and splits, to evaluate the baseline performance of HybridNER on general-domain conventional entity recognition.

#### 2.2.2. Specialized Domain Datasets

(1) CLUENER Dataset

The CLUENER dataset is a fine-grained NER dataset for specialized domains released by the CLUE Benchmark in 2020. Its corpus is derived from THUCNews news snippets with extended annotations and includes 14 entity types such as address, company, position, work, organization, person, book, and film. Sentences exhibit high entity density and contain many long-tail entities, which enables evaluation of a model’s generalization to rare categories. Officially, only the training set with 10,748 sentences and the validation set with 1343 sentences are released (the test set is unlabeled). In this study, we keep the training set unchanged and use the validation set as the test set for evaluation to avoid data leakage.

(2) CMeEE Dataset

The CMeEE dataset is a medical-domain NER dataset released in 2021 as part of CBLUE. The corpus originates from real-world sources such as medical encyclopedias, clinical guidelines, and case descriptions. Entity annotations cover nine major categories including diseases, symptoms, medications, examinations, surgeries or procedures, and anatomical sites, among others, and the dataset exhibits typical domain-specific challenges such as varied surface forms and ambiguous boundaries. The official split mirrors that of CLUENER, with 15,000 sentences for training and 5000 for validation (the test set is unlabeled). In this study, we use the validation set as a substitute test set for evaluation and train only on the official training set to reflect cross-domain generalization capability.

(3) SNCM Dataset

To evaluate the performance of HybridNER in niche specialized domains, we independently constructed SNCM dataset. This dataset centers on jamming and spoofing scenarios in the Global Navigation Satellite System (GNSS) [14] and draws on multiple authoritative sources to ensure comprehensive domain coverage. These sources include policy documents outlining national strategies, specialized monographs defining fundamental theories, peer-reviewed articles and technical patents providing parameters, and industry news reports reflecting real-time dynamics. After de-duplication, denoising, and systematic preprocessing, we obtained a high-quality domain-specific corpus.

Regarding the annotation standards, we adhered to the guiding principle of “events as the core, organizational units as the boundary, equipment and technology as the carriers of actions, and platforms and environments as constraints”. To rigorously implement this principle, we adopted an “Ontology-First” strategy utilizing the Protégé tool to define the semantic schema prior to annotation. The resulting schema comprises core semantic domains such as Adversarial Subjects, Adversarial Events, Countermeasure Equipment, and Operational Environment. To further ensure high annotation quality, we implemented a logic-based validation mechanism using the HermiT reasoner to verify the logical consistency of the ontology. This process enforced constraints such as DisjointClasses to ensure that an entity cannot be simultaneously defined as an Event and Equipment, thereby minimizing semantic ambiguities.

**Figure 2:**
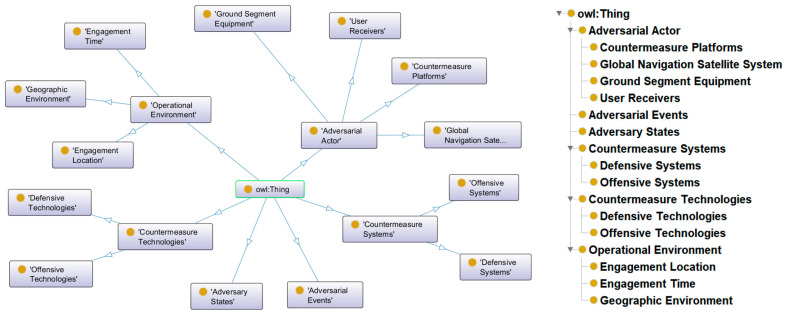
Entity types of the SNCM dataset.

In total, we defined and annotated 13 core entity categories, resulting in a dataset containing 13,439 annotated entities and 7199 relational triples. As visually illustrated in Figure 2 and detailed in Table 1, the dataset exhibits a distinct long-tail distribution, with Countermeasure Platforms and Offensive Systems representing the most prominent classes with 3043 and 2530 entities, respectively. This dataset fills a critical gap in data resources for NER tasks within the GNSS adversarial domain and provides a challenging testbed for evaluating model robustness.

To ensure the objectivity and high quality of the dataset, the annotation task was executed by three postgraduate researchers specializing in GNSS security, who underwent rigorous domain knowledge training prior to the process. We adopted a double-blind independent annotation strategy, where each document was labeled by at least two annotators to minimize subjective bias. Any discrepancies were resolved through a strict adjudication process: conflicting spans were reviewed by a senior supervisor, and final labels were determined based on the predefined ontology constraints and textual evidence, ensuring semantic consistency across the corpus.

### 2.3. Local NER Models

This section introduces the sequence labeling model and span prediction model in the NER task. The sequence labeling models include three variants: BERT-BiLSTM-CRF [15], RoBERTa-BiGRU-CRF [16], and MacBERT-Attn-BiLSTM-CRF [17], all of which have demonstrated excellent performance in the Chinese NER domain. By jointly optimizing deep learning models with Conditional Random Fields (CRF), these models effectively improve the accuracy and robustness of named entity recognition. The span prediction model is based on the SpanNER framework, which not only predicts named entities directly as part of an NER system but also serves as an effective ensemble method, integrating multiple models to enhance overall recognition performance. By predicting the start and end positions of entities, this model reduces the errors associated with traditional methods and has shown high accuracy and flexibility in various NER tasks. This section provides a detailed explanation of the structure, principles, and advantages of these models, highlighting the potential of different models in processing text sequences and offering technical background and methodological support for subsequent NER tasks.

#### 2.3.1. Sequence Labeling Model

The sequence labeling model is used to assign a label to each element in an input sequence and is commonly applied in tasks such as NER, part-of-speech tagging, and sentiment analysis. In this model, the objective is to assign a label to each word or character in the input sequence, thereby mapping the sequence to labels. The core challenge lies in capturing the complex contextual relationships within the input sequence [18]. Compared to traditional independent label prediction methods, sequence labeling models are better at handling dependencies within the sequence. Additionally, by using hierarchical structures like CRF [19], the model can further optimize global prediction accuracy, making it more accurate and robust when handling complex language tasks. This enhances the precision of entity boundary detection and category classification.

(1) BERT-BiLSTM-CRF Model

**Figure 3:**
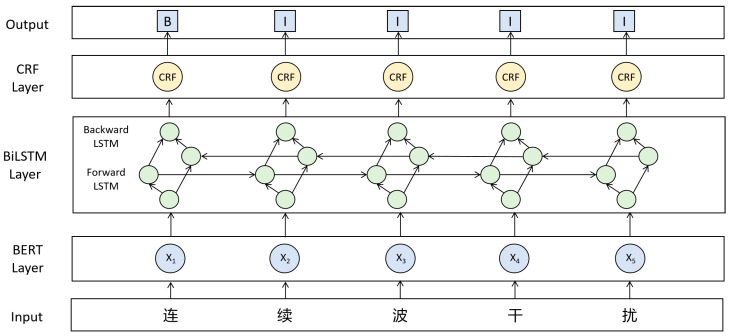
Schematic of the BERT-BiLSTM-CRF model. The Chinese characters shown in the input layer represent the term “Continuous Wave Interference”.

The BERT-BiLSTM-CRF model consists of three modules. The BERT [20] model first encodes the input text sequence, generating context-aware word embedding representations for each word. The BiLSTM [21] is used to capture the bidirectional dependencies within the text, producing a feature vector for each word. The CRF decodes the feature vectors and label sequences based on their conditional probabilities, generating a predicted label sequence. Finally, entities within the sequence are extracted and classified, completing the entire process of Chinese entity recognition. Figure 3 illustrates a schematic of the BERT-BiLSTM-CRF model.

(2) RoBERTa-BiGRU-CRF Model

**Figure 4:**
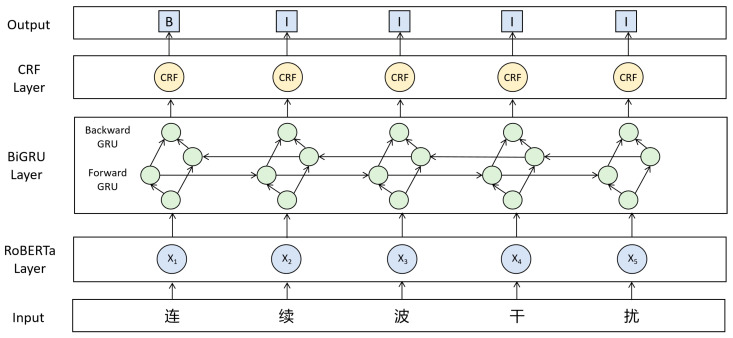
Schematic of the RoBERTa-BiGRU-CRF model. The input layer illustrates a Chinese example sequence meaning “Continuous Wave Interference”.

The RoBERTa-BiGRU-CRF model consists of three modules. RoBERTa [22] provides deep contextual representations for subword sequences. By leveraging a larger unsupervised corpus, a dynamic masking strategy, and removing the “next sentence prediction” task from the pretraining objective, it is able to learn more robust and generalizable semantic features. The BiGRU [23] uses bidirectional gated units to pass information, requiring fewer parameters and converging faster than BiLSTM while still maintaining good modeling capability for long-range dependencies. The CRF layer imposes label transition constraints at the global sequence level and finds the optimal label sequence through a globally normalized decoding process. This allows for the joint determination of entity boundaries and categories, improving the accuracy and robustness of Chinese Named Entity Recognition while ensuring efficiency. Figure 4 illustrates the schematic of the RoBERTa-BiGRU-CRF model.

(3) MacBERT-Attn-BiLSTM-CRF Model

**Figure 5:**
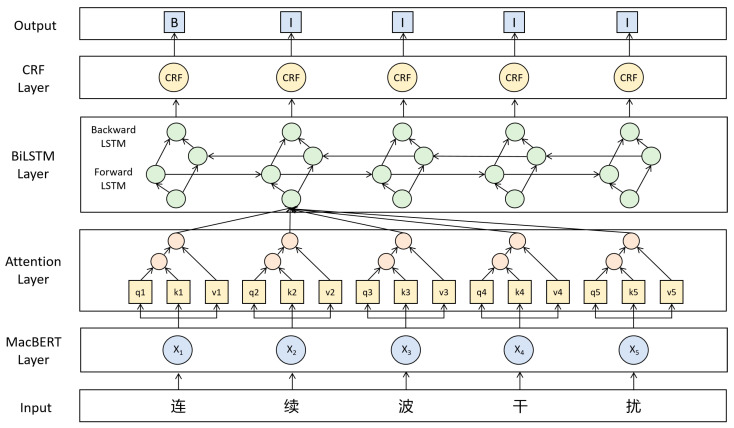
Schematic of the MacBERT-Attn-BiLSTM-CRF model. The non-English terms in the input layer correspond to the phrase “Continuous Wave Interference”.

The MacBERT-Attn-BiLSTM-CRF model consists of four modules. First, MacBERT [24] performs contextual representation learning on the input sequence, introducing a “correction-based masked language modeling” and word-level replacement training strategy. Compared to the standard BERT, this approach effectively mitigates the distribution shift caused by the [MASK] token and enhances robustness to synonymous disturbances and noise in Chinese. The attention mechanism module applies learnable weighting before the features enter the BiLSTM, automatically emphasizing entity trigger words and key dependency segments, which is more effective in suppressing redundant information compared to average pooling or simple convolution. The BiLSTM captures bidirectional long-range semantics and fine-grained syntactic constraints, providing greater stability in cross-word span and boundary determination compared to unidirectional neural networks. The CRF layer constrains the predicted label sequence, ensuring the consistency and coherence of labels. Ultimately, the model achieves high-precision determination of entity boundaries and types, completing the process of Chinese Named Entity Recognition. Figure 5 illustrates the schematic of the MacBERT-Attn-BiLSTM-CRF model.

#### 2.3.2. Span-Based Prediction Model

This study adopts SpanNER as the span prediction model. Compared with token-level labeling, span-based models focus on extracting contiguous text segments, which enables them to better handle noise and heterogeneity in complex open-domain settings. They substantially reduce ambiguity in expressing entity boundaries, improve boundary-decision accuracy, and align more closely with the needs of downstream tasks such as relation extraction [25] and question answering [26].

(1) SpanNER as a Named Entity Recognition System

A span-prediction-based NER framework comprises three principal modules: the token representation layer, the span representation layer, and the span prediction layer.

① Token representation layer

Given a sentence $X=\{x_{1},...,x_{n}\}$, token representations $h_{i}$ are computed as follows:

(1) $$u_{1},...,u_{n}=EMB(x_{1},...,x_{n})$$

(2) $$h_{1},...,h_{n}=BILSTM(u_{1},...,u_{n})$$

Here, EMB denotes contextual embeddings, and BiLSTM denotes a bidirectional long short-term memory network. This layer integrates contextualized embeddings with sequence modeling, providing the foundation for subsequent span representations.

② Span representation layer

We enumerate all possible spans in $X=\{x_{1},...,x_{n}\}$, $S=\{s_{1},...,s_{i},...s_{m}\}$, and assign a label to each span. Let $e_{i}$ and $d_{i}$ be the start and end indices of span $s_{i}$, respectively, so that $s_{i}=\{x_{e_{i}},x_{e_{i}+1},...,x_{d_{i}}\}$. The vector representation of each span is formed by concatenating a boundary embedding with a span-length embedding. The boundary embedding consists of the contextual representations of the span’s start and end tokens, $z_{i}^{e}=[h_{e_{i}};h_{d_{i}}]$, while the length embedding is obtained from a learnable lookup table based on the span length, $z_{i}^{l}$.

The final span representation is $s_{i}=[z_{i}^{e};z_{i}^{l}]$. The span representation layer maps candidate segments into a unified, comparable vector space and explicitly encodes the “boundary + length” features, thereby facilitating subsequent independent scoring and classification.

③ Span prediction layer

For each span $s_{i}$, we perform a softmax classification to obtain the probability of label y:

(3) $$P(y|s_{i})=\frac{score(s_{i},y)}{\sum_{y^{\prime }\in Y}score(s_{i},y^{\prime })},\;score(s_{i},y_{k})=exp(s_{i}^{T}y_{k})$$

where score(·) measures the compatibility between a span and a label, and $y_{k}$ denotes a learnable label embedding. This design replaces traditional CRF-style sequence decoding with span-label matching scores, enabling end-to-end determination of entity types.

(2) SpanNER as a System Integrator

Traditional ensemble methods for NER can be broadly divided into unsupervised and supervised approaches. In unsupervised settings, voting schemes are widely used, including majority voting, voting weighted by the overall F1 score, and voting weighted by per-class F1 scores. In supervised settings, stacking is representative: a higher-level classifier relearns from the outputs of base models, typically instantiated with support vector machines (SVM), random forests (RF), or extreme gradient boosting (XGBoost).

However, conventional ensemble methods face several limitations. First, they often rely heavily on labor-intensive feature engineering and external knowledge, making end-to-end training and cross-domain transfer difficult; they also require retraining base systems and collecting additional training samples via cross-validation to fit the combiner, which undermines practicality. Second, most studies restrict system combination to the sequence-labeling paradigm and thus fail to effectively absorb complementary information from span-prediction architectures. Moreover, base-model training and combiner training are performed independently, making it hard to coordinate within a shared parameter and representation space, which in turn compromises overall consistency and empirical performance.

In contrast, the SpanNER model can assume a dual role: it functions both as the base model for recognizing named entities in text and as a combiner that assigns a score to each entity. Consequently, the approach requires no additional feature engineering, avoids reliance on a single paradigm, and introduces no extra training overhead; both recognition and ensembling are completed under a shared parameterization.

Let s denote a candidate span in the test sentence. Let $L^{\prime }$ be the multiset of labels predicted by the m base systems (so $\left|L^{\prime }\right|=m$), and let L be the NER label set (so $\left|L\right|=c$, where c is the number of entity categories). For each label l∈L, the probability of assigning span s to label l is computed by aggregating the scores of the base systems that predicted l:

(4) $$P(s,l)=\sum_{l^{\prime }\in L^{\prime }⋀l\in L}score(s,l^{\prime })$$

Finally, the predicted label for span s is

(5) $$\underset{l\in L}{\arg\;\max}\;P(s,l)$$

### 2.4. HybridNER Method

This section presents the overall architecture and workflow of HybridNER. By performing a three-stage deep ensemble of multiple models, HybridNER further improves the effectiveness and precision of NER. The subsequent subsections provide a detailed account of the overall pipeline and each step.

#### 2.4.1. Workflow of HybridNER

HybridNER comprises three main steps designed to combine locally deployed models with a LLM, thereby enhancing accuracy and robustness in NER. The procedure is as follows:

(1) The test sentence is first processed by multiple local models for entity recognition, including both sequence-labeling models and span-prediction models. For sequence labeling, the output is $y={[y}_{1},y_{2},...,y_{n}]$, where $y_{i}$ denotes the label of the i-th token. For span prediction, the output is $y=[(x^{e},x^{d},label),u]$, where $x^{e}$ and $x^{d}$ are the start and end positions of the entity span, label is the entity type, and u is the associated uncertainty estimate.
(2) The SpanNER model measures the similarity of predictions via a score function and assigns weights to the outputs of the base models. Concretely, SpanNER first computes the similarity between each base model’s prediction and the annotations, assigns a score to each model, and then performs score-weighted fusion so that more reliable models contribute more. Through this process, SpanNER effectively integrates the complementary strengths of different models, improving the accuracy and stability of the final result. The label with the highest aggregated score is selected as the predicted output, thereby achieving complementary integration and optimization across models.
(3) The uncertainty from the local models is compared against a preset threshold τ. If an entity’s uncertainty exceeds τ, it is deemed uncertain and forwarded to the LLM for further classification; if the uncertainty is below τ, no correction is applied and the local model’s prediction is retained. Finally, the outputs from the local models and the classifications from the LLM are fused to yield the final NER results.

#### 2.4.2. Entity Recognition by Local Models

(1) Predictions from sequence labeling models

Sequence labeling is a discriminative paradigm that learns the mapping between an input token sequence and an output label sequence to predict entities. For comparative analysis, we employ three models: BERT–BiLSTM–CRF, RoBERTa–BiGRU–CRF, and MacBERT–Attn–BiLSTM–CRF. Given an input sentence $x={[x}_{1},x_{2},...,x_{n}]$, where $x_{i}$ denotes the feature vector of the i-th token, the model outputs a label sequence $y={[y}_{1},y_{2},...,y_{n}]$, with $y_{i}$ the label assigned to token i. Labels follow the BIO tagging scheme, where B denotes the beginning of an entity, I denotes tokens inside the entity, and O denotes tokens outside any entity. Accordingly, sequence labeling models output token-level annotations that can be aggregated into task-relevant entity spans.

(2) Predictions from the span-based model

Span-based prediction is a generative modeling approach that estimates the joint probability of the text sequence and the label sequence. In this study, we adopt SpanNER as the span prediction model to infer the start and end positions of entities and assign their types. Given a sentence $x={[x}_{1},x_{2},...,x_{n}]$, the model produces outputs of the form $y=[(x^{e},x^{d},label),u]$, where $x^{e}$ and $x^{d}$ denote the start and end indices of an entity span, label is the entity type, and u is an uncertainty probability derived from the model’s confidence distribution. By analyzing inter-token dependencies, the span-based model predicts plausible entity boundaries and attaches an uncertainty probability to each prediction to quantify reliability. This approach is advantageous for handling overlapping and nested entities, better accommodating complex NER scenarios; moreover, by directly predicting span boundaries, it can reduce computational overhead in certain cases.

#### 2.4.3. Ensemble of Multiple Local Models

Assume there are m base systems to be ensembled, and let L denote the predefined NER label set (e.g., “PER/LOC/ORG/O” in MSRA). The ensemble proceeds in four steps:

(1) Collect outputs from the base systems

Obtain, for the same candidate span *s*, the multiset of predicted labels $L^{\prime }$ from all m base systems (so $\left|L^{\prime }\right|=m$). For example:

Base System 1: BERT-BiLSTM-CRF (sequence labeling) predicts s = “Shang Hai” as “LOC”.

Base System 2: SpanNER (span-based model) predicts s = “Shang Hai” as “LOC”.

Base System 3: RoBERTa-BiGRU-CRF (sequence labeling) predicts s = “Shang Hai” as “ORG”.

Base System 4: MacBERT-Attn-BiLSTM-CRF (sequence labeling) predicts s =“Shang Hai” as “O”.

Thus $L^{\prime }\;$= {“LOC”,“LOC”,“ORG”,“O”}.

(2) Compute span–label compatibility scores

Using the score function from the SpanNER span-prediction layer, compute the compatibility between span *s* and each predicted label $l^{\prime }$:

(6) $$score(s,l^{\prime })=exp(s^{T}\cdot {l^{\prime }}_{k})$$

where ${l^{\prime }}_{k}$ is the learnable vector associated with label $l^{\prime }$. Here, $s^{T}\cdot {l^{\prime }}_{k}$ denotes the inner product between the span vector s and the label vector ${l^{\prime }}_{k}$; the exponential maps the result to a positive value. A higher score indicates a stronger match between the label and the span.

(3) Aggregate scores by label category

Group the m predictions by their label and sum the corresponding compatibility scores to obtain, for each l∈L, the aggregated probability is $P(s,l)=\sum_{l^{\prime }\in L‘⋀l\in L}score(s,l^{\prime })$. For the running example:

$P(s,“LOC”)={score(s,“LOC”)}_{1}+{score(s,“LOC”)}_{2}$;

$P(s,“ORG”)={score(s,“ORG”)}_{3}$;

$P(s,“O”)={score(s,“O”)}_{4}$.

(4) Determine the final predicted label

Select the label with the highest aggregated probability as the final output of the SpanNER-based ensemble:

(7) $$\underset{l\in L}{\arg\;\max}\;P(s,l)$$

For example, if P(s,“LOC”) is the largest, the final prediction for s is LOC.

#### 2.4.4. Uncertainty Estimation and Filtering Mechanism

To ensure the reliability of the entity recognition results and optimize the interaction with the LLM, we introduce a rigorous uncertainty estimation mechanism based on the SpanNER architecture.

(1) Mathematical Formulation of Uncertainty

As described in Section 2.3.2, the span prediction layer computes the probability distribution of labels for a candidate span $s_{i}$ using the Softmax function. Let $P(y|s_{i})$ denote the probability of assigning label y to span $s_{i}$. We define the confidence score $C(s_{i})$ as the maximum probability in the distribution:

(8) $$C(s_{i})=max\;P(y|s_{i})=max\;\frac{exp(s_{i}^{T}y)}{\sum_{y^{\prime }\in Y}exp(s_{i}^{T}y^{\prime })}$$

Consequently, the uncertainty $score\;u(s_{i})$ is quantified as the complement of the confidence score:

(9) $$u(s_{i})=1-C(s_{i})$$

This metric $u(s_{i})\in [0,1]$ serves as a direct indicator of the model’s hesitation. A higher $u(s_{i})$ implies that the local model struggles to distinguish between entity types or between an entity and a non-entity background.

(2) Threshold Selection and Calibration

The selection of the uncertainty threshold τ is critical for balancing system performance and computational cost. A lower threshold would route more entities to the LLM, potentially improving recall but significantly increasing inference latency and token consumption. Conversely, a higher threshold relies more on local models, which may lack domain-specific knowledge. We continuously conduct empirical analysis on the validation set to calibrate this parameter, treating entities exceeding τ as “high-risk” and routing them to the LLM for a second-stage verification. This strategy ensures that the LLM is engaged only when the local ensemble is demonstrably unreliable, thereby maximizing the marginal gain of the external knowledge injection.

#### 2.4.5. Integration of Local Models with Large Language Models

**Figure 6:**
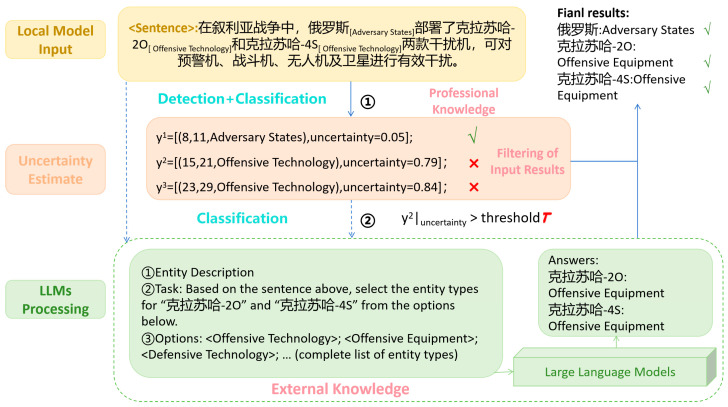
Schematic of integrating local models with large language models. The Chinese input text serves as a domain-specific example, translating to: “In the Syrian war, Russia deployed Krasukha-20 and Krasukha-4S jammers…”. The identified entities correspond to “Russia” (Adversary States) and “Krasukha-20/4S” (Offensive Systems).

This section describes how integrating local models with LLMs can improve performance on NER. NER requires models to effectively capture the task-specific data distribution of entities. However, general-purpose LLMs—such as GPT [27], Llama [28], and DeepSeek [29]—are not explicitly optimized for information extraction. Prior studies have shown that these models exhibit notable shortcomings when handling highly specialized entity types. These limitations stem from the lack of NER-specific specialization, and directly applying LLMs to entity recognition often leads to suboptimal results. To address this issue, we link local models with LLMs and enable collaborative inference between a locally fine-tuned model and a black-box LLM [30], thereby enhancing overall NER performance. The integration workflow is illustrated in Figure 6.

(1) Initial recognition by local models

Each local model first performs entity recognition to identify candidate entities in the current text. The SpanNER model then applies its system-level ensembling capability to combine the multiple local models and produce a preliminary result. At this stage, SpanNER assigns an uncertainty value to each recognized entity, indicating the model’s confidence. Entities with high uncertainty are forwarded to the LLM for further classification and verification.

(2) Uncertainty-based routing

Next, entities deemed unreliable are detected using the uncertainty probabilities output by the local model. If an entity’s uncertainty exceeds a preset threshold, it is passed to the LLM for second-stage classification, thereby preventing the final decision from being dominated by uncertain local predictions [31].

(3) Second-stage classification by the LLM

The LLM receives entities from the local model and, leveraging its extensive knowledge and reasoning ability, adapts to previously unseen entities and provides additional classification capacity. This reframes the LLM’s role from entity identification to label selection, which better exploits the strengths of large models and compensates for the local models’ limitations on specialized entities.

For example, when processing the toponym “Shang Hai”: if the local model recognizes the entity as “LOC” with a low uncertainty value, it directly outputs the result. Conversely, if the local model is uncertain and yields a high uncertainty value, “Shang Hai” is routed to the LLM. Upon receiving the entity, the LLM, based on its learned knowledge, further confirms the entity type and makes a more accurate determination. Through this collaboration, the two components complement each other: local models quickly and effectively handle simple entities, while the LLM provides additional classification capability for complex cases, thereby improving the precision and robustness of NER.

## 3. Results

### 3.1. Experimental Setup

To comprehensively evaluate the performance of HybridNER as a system combiner, we designed a rigorous validation protocol involving four diverse datasets: the general-domain MSRA benchmark and three specialized-domain datasets, specifically CLUENER, CMeEE, and the self-constructed SNCM. We adopted standard Information Extraction metrics—Precision (P), Recall (R), and F1-score—to quantify model performance. In terms of baselines, we systematically compared HybridNER against three representative unsupervised ensemble methods: Majority Voting (VM), Voting Weighted by Overall F1 (VOF1), and Voting Weighted by Class-wise F1 (VCF1). To ensure a fair comparison and simulate realistic testing conditions, we employed a five-fold cross-validation strategy on the training set to generate out-of-fold predictions for all base models. Furthermore, to mitigate experimental uncertainty and verify the stability of our improvements, we conducted multiple independent runs and assessed statistical significance using the Wilcoxon signed-rank test.

In this study, we employed the DeepSeek-V3 model (accessed via API) as the collaborative large language model component. DeepSeek-V3 was selected for its advanced reasoning capabilities in complex linguistic tasks and its highly competitive cost-performance ratio. To ensure the reproducibility of our experiments and minimize generation randomness, we configured the API with the following parameters: temperature was set to 0, top_p was set to 1.0, and the maximum output length was restricted to 128. These settings force the model to behave deterministically, ensuring that the classification of high-uncertainty entities remains consistent across multiple runs. We designed a structured prompt template to constrain the LLM’s output strictly to the predefined label set, preventing open-ended generation or hallucinations. The detailed prompt template and the instruction format used in this experiment are provided in Appendix A.

### 3.2. Baseline Methods

(1) Majority Voting (VM)

All taggers are assigned equal weights; the final prediction is determined by the number of votes each class receives [32]. In the event of a tie, we resolve it by consulting class prior frequencies estimated from the training or validation data. For sequence labeling, voting is performed at the tagging unit level, and constrained decoding is applied to eliminate transitions that violate the tagging scheme. This approach has low implementation cost and exhibits some robustness to single-point errors, making it a representative ensemble baseline.

(2) Voting weighted by overall F1 (VOF1)

This method characterizes each tagger’s global reliability by its overall F1 score on the validation set [33]. The score is monotonically mapped and normalized to yield a single, class-agnostic weight. For sequence labeling, each tagger casts a weighted vote only for the class it proposes, and the class with the highest accumulated weight is selected as the output; tie-breaking follows the same rule as Majority Voting. The method assumes limited relative performance variation across classes, and thus offers a simple implementation with comparatively low variance.

(3) Voting Weighted by Class-wise F1 (VCF1)

This scheme first computes per-class F1 scores on the validation set and then applies a class-level monotonic mapping and normalization to obtain weights that vary across classes [34]. For sequence labeling, only the effective weights corresponding to the candidate class are accumulated, thereby granting greater decision influence to taggers that perform better on that specific class; tie handling follows the rules described above. To improve robustness for long-tail classes, one may smooth the scores of classes with low support or fall back to global weights when samples are scarce. This method is particularly suitable when class imbalance is pronounced or model strengths are complementary across classes, though it requires more fine-grained weight estimation and reporting than VOF1.

In summary, the system combiners used in the baselines incur higher computational and engineering costs: during data collection they require five-fold cross-validation on the original training set to obtain out-of-fold predictions, upon which the subsequent system combination is performed.

### 3.3. Comparative Experiments

For the NER task, we evaluate four datasets spanning different domains and label taxonomies: the general-domain MSRA and three specialized-domain datasets—CLUENER, CMeEE, and SNCM. We compare recognition accuracy and overall effectiveness under four combination strategies: Majority Voting (VM), Voting weighted by overall F1 (VOF1), Voting weighted by class-wise F1 (VCF1), and HybridNER. The results are summarized in Table 2.

From Table 2, it can be observed that the HybridNER method outperforms Majority Voting, Weighted Voting Based on Overall F1, and Weighted Voting Based on Class-wise F1 across all datasets. Specifically, the method achieves an improvement of 1.35% on the general-domain MSRA dataset, 6.42% on the specialized-domain CLUENER dataset, 6.27% on CMeEE, and 6.42% on the SNCM dataset. The significant performance improvement in specialized datasets is attributed to the ability of large models to complement these domains with more domain-specific knowledge, which is more evident than in the general-domain datasets. Lastly, HybridNER demonstrates a notable improvement in recall across all datasets, with the large model effectively reclassifying entities that local models either failed to recognize or misclassified as non-entities, thereby significantly boosting recall and aligning with the core requirement of NER tasks in open environments: to cover more valid entities while controlling classification errors. The specific experimental results are analyzed as follows:

(1) HybridNER outperforms Majority Voting, Weighted Voting Based on Overall F1, and Weighted Voting Based on Class-wise F1 across all datasets. This can be attributed to HybridNER’s ability to integrate the strengths of multiple local models through a weighted strategy, effectively combining the strengths and weaknesses of each model. The multi-model collaboration strategy allows the system to choose the most appropriate model output for different entity categories, reducing the risk of overfitting or bias inherent in using a single model. Moreover, HybridNER not only performs well on existing datasets but also exhibits excellent scalability. As the number of datasets increases, especially with the addition of specialized-domain datasets, HybridNER can flexibly integrate new local models and adjust the usage strategy of the large language model, enabling it to adapt to the evolving needs of entity recognition.
(2) Although HybridNER performs excellently in the general domain, its performance improvement is particularly pronounced in specialized domains. By combining local models with large language models, HybridNER significantly improves the recognition of specialized terms and rare entities in professional domains. In the SNCM dataset, the base models tend to overfit or exhibit instability, while HybridNER, relying on multi-model integration and an uncertainty-based decision mechanism, demonstrates strong adaptability and robustness, maintaining stable performance in complex and dynamic environments.
(3) In named entity recognition tasks, precision and recall typically exhibit an inverse relationship, with the F1 score seeking a balance between the two. HybridNER achieves dual improvements in recall and F1 score by utilizing large language models as an external knowledge base. Leveraging the vast training data and reasoning ability of these models, HybridNER compensates for the limitations of local models in specialized entity recognition, leading to a significant increase in recall. In each entity recognition decision process, HybridNER evaluates uncertainty probabilities to determine whether to submit an entity to the large language model for further confirmation. This uncertainty-based decision-making approach allows the method to maintain high precision while avoiding the pitfalls of traditional models, which may either be too conservative or overly aggressive, missing important entities. As a result, the overall F1 score is improved. Compared to Majority Voting and Weighted Voting methods, this innovative strategy in HybridNER demonstrates superior performance in balancing precision and recall.

### 3.4. Ablation Experiments

To comprehensively validate the effectiveness of the proposed HybridNER framework and quantify the contributions of its constituent modules, we conducted a systematic ablation study. We compared HybridNER against two distinct categories of baselines comprising individual component models, which represent sequence labeling and span-prediction paradigms, as well as a Pure LLM-based extraction baseline utilizing DeepSeek-V3 in a zero-shot setting.

The results, shown in Table 3, indicate that HybridNER achieves the highest F1 score across all four datasets: 97.47 for MSRA, 83.34 for CLUENER, 77.01 for CMeEE, and 81.59 for SNCM. These improvements represent increases of 1.85, 1.68, 1.48, and 1.81 percentage points, respectively, over the strongest baseline. The performance boost balances both precision and coverage while demonstrating robustness to datasets with more granular categories and higher noise levels. These results suggest that hybrid modeling effectively combines the strengths of two paradigms: the sequence labeling branch captures local context and label dependencies, while the span-based branch performs global boundary validation and type classification. The two components work synergistically to reduce boundary errors and type conflicts. Furthermore, degrading the model to a single paradigm leads to a drop in F1 scores ranging from 1.4 to 7.9, confirming the necessity of each module and demonstrating the generalization and robustness of the proposed framework for multi-domain NER tasks.

In terms of the comparison with the Pure LLM baseline, the experimental results indicate that HybridNER consistently outperforms the standalone DeepSeek-V3 model across all datasets. Qualitative analysis reveals that while the Pure LLM demonstrates commendable recall by benefiting from its extensive open-world knowledge, it frequently exhibits limitations in precise boundary positioning. Consistent with recent findings in generative information extraction, large language models lack the explicit inductive bias for span boundary detection found in sequence labeling models. Consequently, the pure generative approach tends to erroneously include punctuation or adjacent modifiers within entity spans or fail to strictly adhere to the token-level annotation schema, leading to a marked degradation in precision. HybridNER effectively mitigates these issues by leveraging the specialized boundary detection capabilities of local models to filter candidate spans.

### 3.5. Efficiency and Cost Analysis

A critical challenge in deploying LLMs for information extraction is the significant operational overhead, including high inference latency and API costs. Processing every sentence through a large model is often impractical for real-time applications. Our HybridNER framework addresses this by acting as a high-precision filter, routing only entities with high uncertainty to the LLM. Based on our experimental statistics, the local SpanNER model successfully handles approximately 70% to 75% of the entity recognition tasks. Consequently, the LLM is invoked for only a small fraction of the total workload. In terms of financial cost, using the current official pricing of the DeepSeek-V3 API (Input: $0.14/1 million tokens), the operational cost of HybridNER is negligible compared to a full-LLM approach. Furthermore, regarding latency, local model inference typically operates on the order of milliseconds, whereas API-based LLM calls involve network and generation latency often exceeding one second. By restricting LLM usage to only the most difficult cases, HybridNER effectively reduces the system-wide average latency by nearly an order of magnitude. This architecture achieves a “Pareto optimal” balance, retaining the reasoning power of Large Language Models while maintaining the high throughput characteristic of lightweight local models.

## 4. Discussion

This paper proposes HybridNER, a hybrid NER method that integrates local models with large language models. Through deep multi-model integration and an uncertainty-driven collaborative mechanism, HybridNER effectively enhances the performance and robustness of NER tasks in both general and specialized domains. The main conclusions of this research are as detailed below.

Firstly, HybridNER establishes a two-layer architecture that integrates local model ensembles with second-stage optimization by large language models, complementing the advantages of different paradigm models. At the local model layer, this method innovatively combines sequence labeling models with span-based prediction models. By calculating span-label compatibility scores and aggregating similar label scores, it dynamically weights the outputs of each model. This approach retains the sequence labeling model’s ability to capture context-dependent relationships while leveraging the span-based model’s strengths in recognizing entity boundaries and nested or overlapping entities, thus addressing boundary errors and type conflicts that single models often encounter in complex texts. Secondly, HybridNER introduces a decision-making mechanism based on uncertainty thresholds, enabling efficient resource allocation and precise control over recognition accuracy. The method compares the uncertainty probabilities of local model outputs with preset thresholds, only passing high-uncertainty entities to the large language model for secondary classification. Qualitative analysis indicates that HybridNER primarily benefits ambiguous and long-tail categories, effectively correcting ‘knowledge-intensive’ errors where local models struggle due to limited supervision. Conversely, for highly structured and frequent entities, local models already achieve near-optimal performance, yielding minimal marginal gains from LLM intervention. This highlights the framework’s targeted effectiveness in addressing low-resource and context-dependent recognition challenges. Finally, multi-domain experiments validate the generalizability and robustness of HybridNER. The method outperforms traditional ensemble methods, such as majority voting and weighted voting, in terms of F1 scores on both general and specialized datasets. The performance improvement is significantly higher in specialized domains compared to general domains, demonstrating its ability to effectively adapt to complex, diverse professional scenarios. Furthermore, ablation experiments show that removing the sequence labeling or span prediction modules leads to a decline in F1 scores, further confirming the necessity of each component and the overall framework’s rationality. This provides a solution that balances both accuracy and efficiency for multi-domain NER tasks.

Based on the HybridNER method proposed in this study, future work will focus on three main areas of deep optimization to further improve its performance and applicability in NER tasks. First, an adaptive threshold decision model will be developed to replace the current fixed preset threshold mechanism. By dynamically adjusting uncertainty determination standards based on the text feature differences in different domains, entity category distributions, and real-time feedback from the recognition results, the system can balance recognition accuracy with resource consumption efficiency while avoiding the misclassification of high-value entities and reducing redundant calls to the large language model. Second, domain-adaptive learning strategies will be introduced to enhance cross-domain transferability. For low-resource and highly specialized emerging domains, fine-tuning local model parameters with a small number of domain-specific samples and incorporating domain-specific knowledge prompts into the large language model interaction process will improve the system’s ability to recognize niche domain entities and expand HybridNER’s application scope. Finally, efforts will be made to streamline and optimize the method for greater efficiency. To address the computational cost introduced by multi-model integration and large language model calls, model compression techniques will be employed to reduce the size of local model parameters. Simplified prompt templates will be designed to optimize large language model interactions while exploring incremental learning mechanisms that allow the system to continuously absorb new domain knowledge, facilitating performance iteration and better adapting to the dynamic, changing needs of real-world applications.

## Figures and Tables

**Table 1:** Statistics of Entity Types in the SNCM Dataset.

Entity Category	Count	Entity Category	Count
Countermeasure Platforms	3043	Offensive Systems	2530
Adversary States	1458	Offensive Technologies	1119
Engagement Time	1074	Defensive Technologies	767
Defensive Systems	671	Adversarial Events	656
Ground Segment Equipment	594	Geographic Environment	489
Engagement Location	362	Global Navigation Satellite System	347
User Receivers	329	Total Entities	13,439

**Table 2:** Performance of system combination methods across datasets.

Dataset	Combination Method	P	R	F1
MSRA Dataset	Majority Voting	97.41	93.64	95.49
	Voting weighted by overall F1	96.78	95.48	96.12
	Voting Weighted by Class-wise F1	97.32	94.49	95.89
	**HybridNER Method**	**98.44**	**96.52**	**97.47**
CLUENER Dataset	Majority Voting	80.53	73.39	76.80
	Voting weighted by overall F1	83.49	70.86	76.66
	Voting Weighted by Class-wise F1	82.91	72.16	76.92
	**HybridNER Method**	**83.83**	**82.87**	**83.34**
CMeEE Dataset	Majority Voting	73.13	68.31	70.64
	Voting weighted by overall F1	73.53	68.15	70.74
	Voting Weighted by Class-wise F1	73.28	68.31	70.71
	**HybridNER Method**	**77.14**	**76.89**	**77.01**
SNCM Dataset	Majority Voting	76.95	73.46	75.17
	Voting weighted by overall F1	76.95	73.46	75.17
	Voting Weighted by Class-wise F1	77.35	71.84	74.50
	**HybridNER Method**	**82.76**	**80.45**	**81.59**

**Table 3:** Performance of Base Models on Datasets.

Dataset	Model	P	R	F1
MSRA Dataset	BERT-BiLSTM-CRF	94.99	94.15	94.56
	RoBERTa-BiGRU-CRF	96.26	94.30	95.27
	MacBERT-BiLSTM-Attention-CRF	95.47	95.76	95.62
	SpanNER	94.57	92.54	93.54
	**HybridNER Method**	**98.44**	**96.52**	**97.47**
CLUENER Dataset	BERT-BiLSTM-CRF	70.55	76.14	73.24
	RoBERTa-BiGRU-CRF	72.84	76.26	74.51
	MacBERT-BiLSTM-Attention-CRF	70.78	75.63	73.13
	SpanNER	81.59	81.73	81.66
	**HybridNER Method**	**83.83**	**82.87**	**83.34**
CMeEE Dataset	BERT-BiLSTM-CRF	68.42	71.34	69.85
	RoBERTa-BiGRU-CRF	69.49	71.82	70.64
	MacBERT-BiLSTM-Attention-CRF	68.76	72.15	70.41
	SpanNER	75.39	75.67	75.53
	**HybridNER Method**	**77.14**	**76.89**	**77.01**
SNCM Dataset	BERT-BiLSTM-CRF	75.60	74.70	75.15
	RoBERTa-BiGRU-CRF	73.18	74.70	73.93
	MacBERT-BiLSTM-Attention-CRF	72.89	74.40	73.64
	SpanNER	81.25	78.37	79.78
	**HybridNER Method**	**82.76**	**80.45**	**81.59**

## Data Availability

The original contributions presented in this study are included in the article. Further inquiries can be directed to the corresponding author.

## Appendix A

To ensure the scalability of HybridNER across diverse scenarios—ranging from general domains to specialized fields—we designed a unified, structure-constrained prompt template. As detailed in Table A1, the template utilizes a Slot-Filling Mechanism. During the inference phase, the domain-specific slots (denoted by brackets {}) are dynamically populated based on the target dataset. For instance, when processing the CMeEE dataset, the {Target_Domain} slot is instantiated as “Clinical Medicine and Disease Diagnosis,” and the {Candidate_Label_Set} is populated with medical categories (e.g., Disease, Symptom, Procedure). This standardized approach enforces strict semantic constraints on the Large Language Model (DeepSeek-V3), ensuring that the output logic remains consistent regardless of the domain specificity.

**Table A1:** The universal structured prompt template used in the proposed framework.

Prompt Component	Instruction Template
**System Meta-Instruction**	You are a specialized Knowledge Graph Expert in the field of {Target_Domain}. Your objective is to perform precise Named Entity Classification based on the provided taxonomy.
**Task Definition**	Analyze the linguistic context of the input sentence and determine the semantic category of the target text span enclosed in brackets. You must perform the classification strictly according to the domain knowledge of {Target_Domain}.
**Constraint Checklist**	1. Validity Check: If the target span is a generic term, a verb, or does not semantically fit any category in the label set, return “Non-Entity”. 2. Constrained Selection: Select ONE category strictly from the provided {Candidate_Label_Set}. Do not generate labels outside this list. 3. Deterministic Output: Output ONLY the category name. Do not provide definitions, explanations, or punctuation.
**Input Context**	Sentence: “{Input_Sentence}”
**Taxonomy Constraint**	Target Span: “{Entity_Span}”
**Expected Response**	Candidate Label Set: “Candidate_Label_Set”
